# Locked Intramedullary Total Wrist Arthrodesis: A Report of Three Patients With Distal Screw Migration

**DOI:** 10.7759/cureus.27420

**Published:** 2022-07-28

**Authors:** Amir R Kachooei, Christopher M Jones, Pedro Beredjiklian

**Affiliations:** 1 Orthopaedics, Rothman Institute, Thomas Jefferson University, Philadelphia, USA

**Keywords:** implate, distal screw migration, intramedullary locked nail, arthrodesis, wrist

## Abstract

Intramedullary (IM) fixation for the total wrist arthrodesis (TWA) is supposed to lower the hardware complication rate by eliminating soft tissue irritation. In this report, we present three patients with distal metacarpal screw migration requiring unplanned secondary surgery for screw removal in one patient while it was managed nonoperatively in the other two. All three patients had complete radiocarpal fusion by four months postop. There was no attempted third carpometacarpal (CMC) fusion in any of our patients. Screw migration was found between 1.5-3.5 months postop and remained stable until the final follow-up in the nonoperatively managed patients. One patient with screw removal continued to have mild tenderness over the third CMC, which was managed nonoperatively.

## Introduction

Symptomatic end-stage wrist arthritis recalcitrant to conservative measures is often treated with total wrist arthrodesis (TWA) to address pain, weakness, and function. TWA is commonly performed using a dorsal plate or intramedullary (IM) fixation. Although plates provide rigid fixation and acceptable fusion rates, plate-related complications and soft tissue irritation requiring plate removal are between 11% and 16% [1,2]. IM fixation has been used in patients with a poor soft tissue envelope, particularly patients with rheumatoid arthritis, using Steinmann Pins; however, it does not provide rotational stability, which may lead to pseudoarthrosis and pin migration [3,4]. The IMPLATE^®^ (Skeletal Dynamics, Miami, FL) is an IM nail device developed to address hardware prominence and soft tissue irritation while providing comparable stability [5]. Unlike plates, this device offers the versatility of adjusting wrist flexion-extension and radioulnar deviation using a modular connector in different lengths and angles, which connects the proximal and distal IM nails [5]. The proximal nail in the radius is stabilized with three bicortical screws. The distal nail is stabilized in the third metacarpal IM canal using a unicortical screw that compresses the nail against the dorsal inner metacarpal cortex, providing longitudinal and rotational stability. This screw threads into the nail and has a broad flat head to distribute the load and minimize hardware prominence. Theoretically, this device should eliminate complications of soft tissue irritation from prominent hardware, yet there are still hardware-related problems that might require unplanned secondary surgery. In this case report, we present three patients who underwent TWA using the IMPLATE IM nail in whom the distal locking screw backed out.

## Case presentation

Case 1

**Figure 1:**
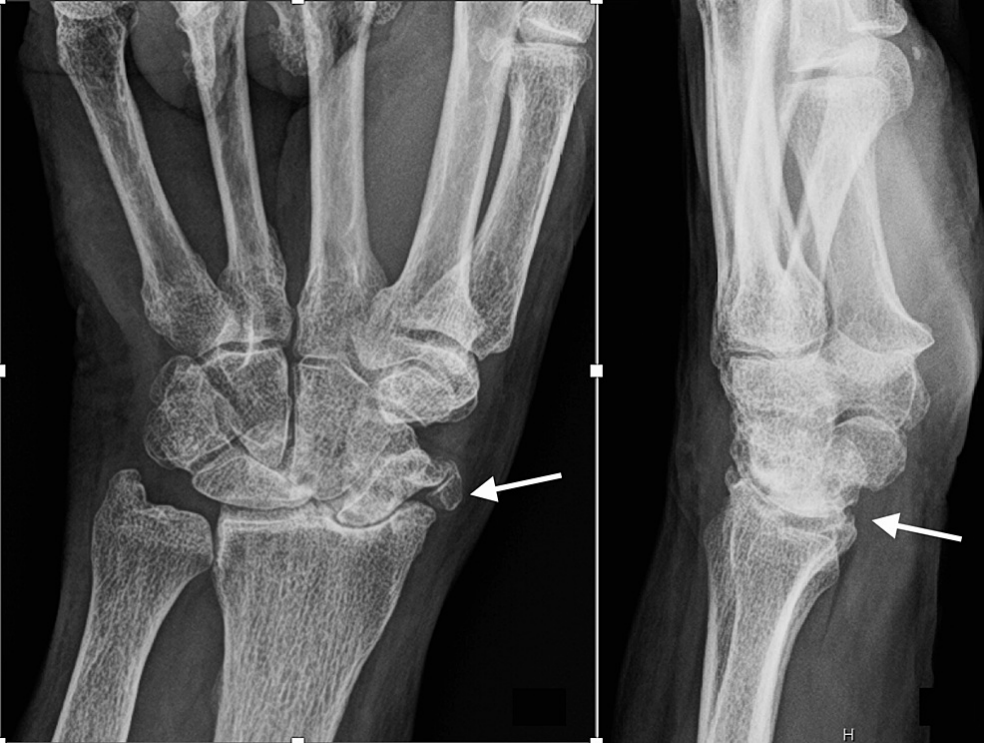
Preoperative radiographs of Case 1 Posteroanterior and lateral views of a 78-year-old woman with chronic post-traumatic scapholunate advanced collapse (SLAC). The white arrows show radiocarpal arthritis.

A 78-year-old retired woman, right-hand dominant, nonsmoker, nondiabetic with an unremarkable past medical history presented with progressive severe left wrist pain, limitation of motion, and weakness due to chronic post-traumatic scapholunate advanced collapse (SLAC) (Figure 1).

**Figure 2:**
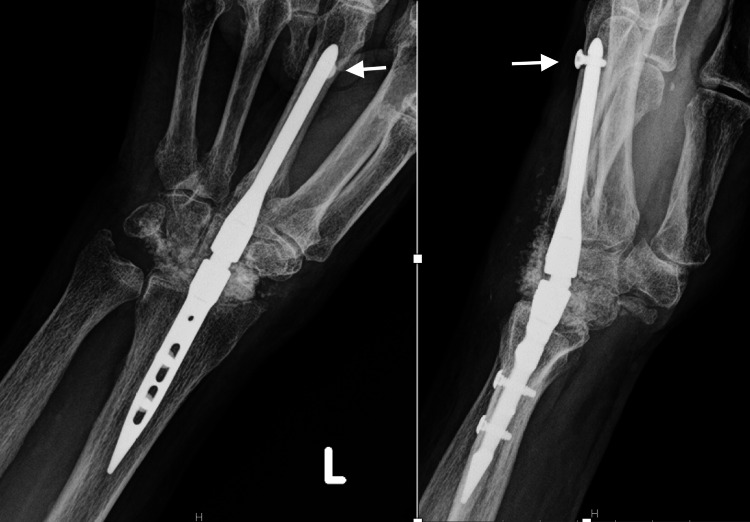
Immediate postoperative radiographs of Case 1 Posteroanterior and lateral views after total wrist arthrodesis using IMPLATE intramedullary nail and proximal row carpectomy. The white arrows show the proper positioning of the distal screw.

She underwent TWA using the IMPLATE intramedullary nail in April 2021 (Figure 2).

**Figure 3:**
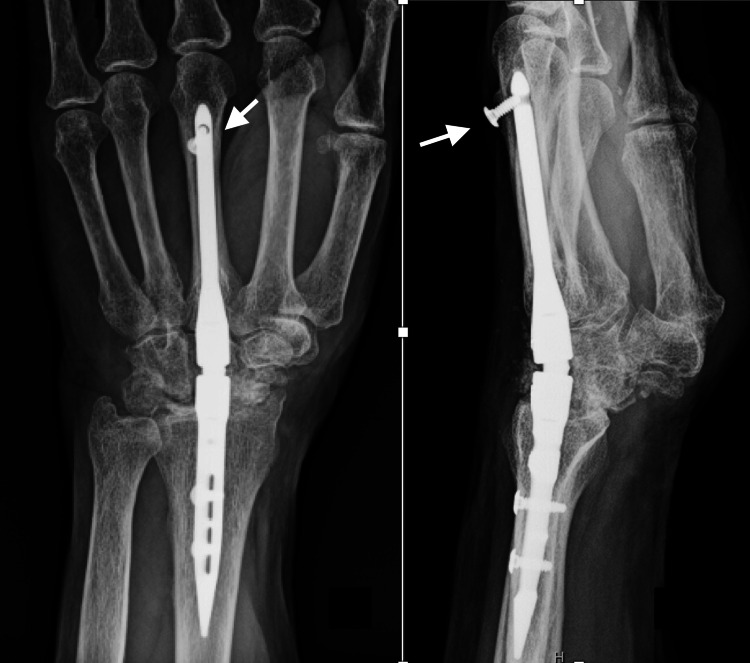
Follow-up radiographs of Case 1 Posteroanterior and lateral views at four months postop show that the distal unicortical screw backed out. The white arrows show the distal screw loosening and migration.

The surgery also included a proximal row carpectomy, bone grafting using morselized bone from the proximal row, and posterior interosseous neurectomy with no attempt to fuse the third CMC joint. She was placed in a custom fabricated wrist splint postoperatively for four months, and physical therapy for finger motions and forearm rotation was started at two months postop in the splint. At four months postop, the radiocarpal joint showed complete fusion on the plain radiographs. At this point, the distal interlocking screw started to back out, identified by radiographs. It became painful and tender six months postop requiring unplanned screw removal surgery (Figure 3).

Her dorsal hand symptoms resolved immediately after screw removal. At five months post screw removal, the third CMC joint remained mildly tender, which was managed nonoperatively.

Case 2

**Figure 4:**
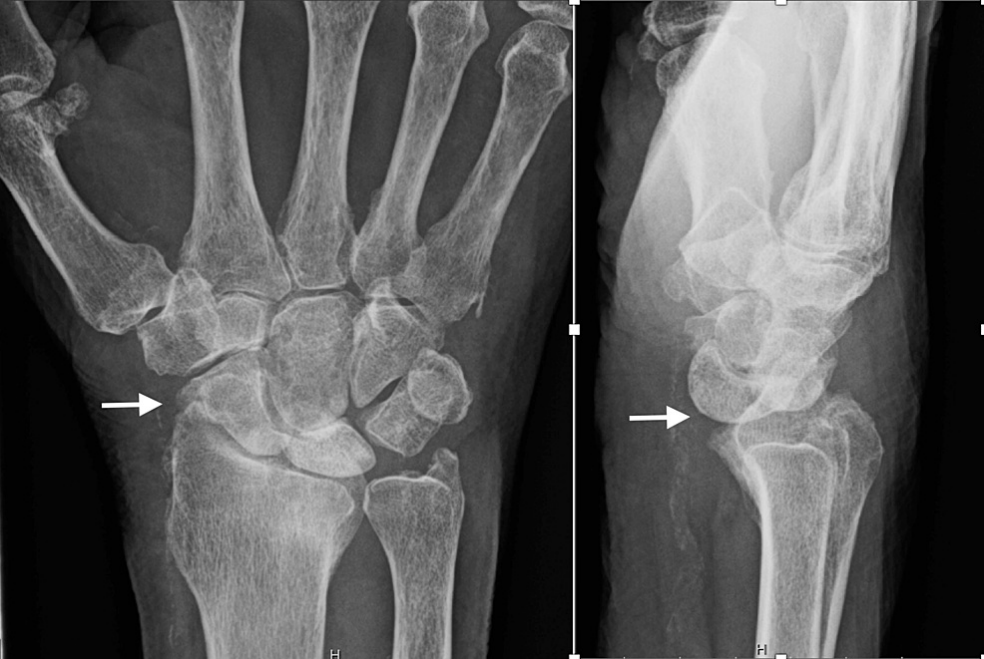
Preoperative radiographs of Case 2 A 90-year-old male with volar lunate subluxation with severe radiocarpal and midcarpal arthritis. The white arrows show the radiocarpal and midcarpal arthritis.

A healthy and active 90-year-old, right-hand dominant, nonsmoker male presented with chronic progressive right wrist pain, weakness, and limited motion. He had a history of successful carpal tunnel release about 15 years prior and trauma to the right upper extremity due to falls. Wrist radiographs showed volar lunate subluxation with severe radiocarpal and midcarpal arthritis (Figure 4).

**Figure 5:**
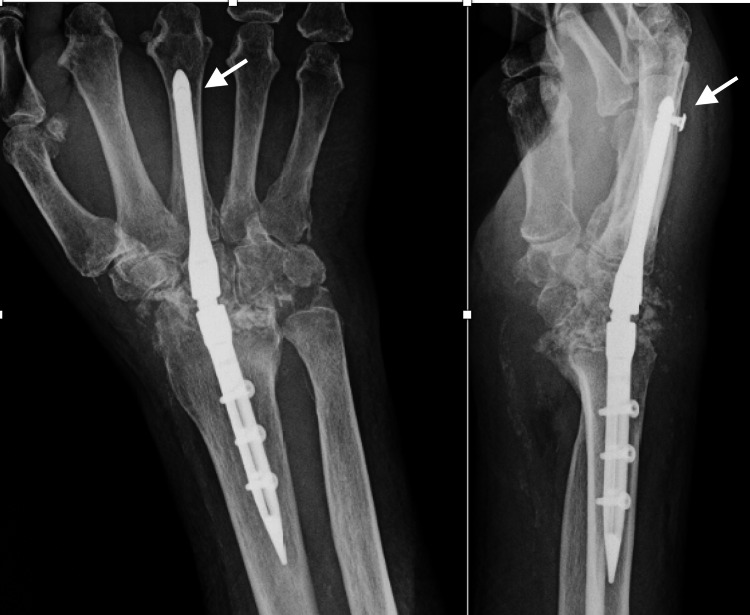
Immediate postoperative radiographs of Case 2 Posteroanterior and lateral views after total wrist arthrodesis using IMPLATE intramedullary nail and proximal row carpectomy. The white arrows show the proper positioning of the distal screw.

MRI showed erosive changes consistent with inflammatory arthritis, though there was no history of serologic markers for inflammatory arthritis. After exhausting nonoperative management, the patient underwent TWA using IMPLATE, revision carpal tunnel release with hypothenar fat pad interposition, and posterior interosseous neurectomy with no attempted fusion of the third CMC joint in September 2020 with no remarkable complication (Figure 5).

**Figure 6:**
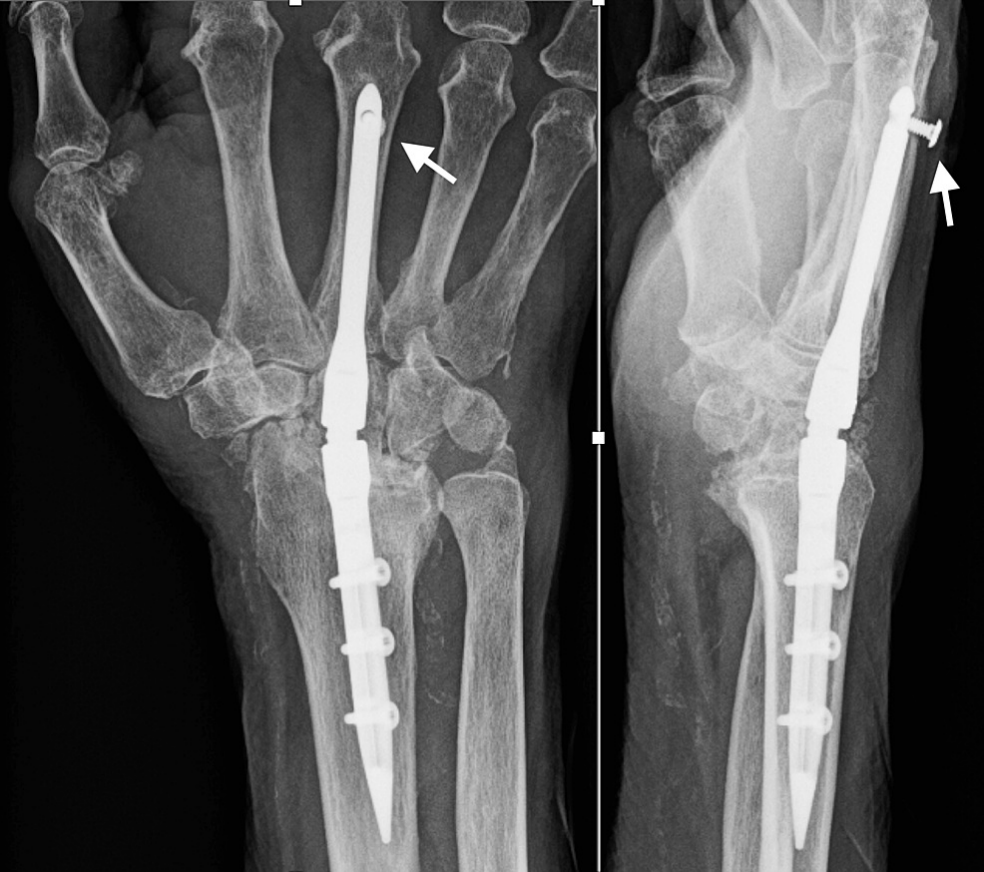
Follow-up radiographs of Case 2 Posteroanterior and lateral views at 1.5 months postop show that the distal unicortical screw backed out. The white arrows show the distal screw loosening and migration.

The gap was filled with morselized bone from the proximal row carpectomy plus allograft bone putty. The wrist was immobilized in a cast postoperatively for two months, and physical therapy for finger motion was started at one month postop in the cast. At 1.5 months postop, the radiographic assessment revealed asymptomatic backing out of the distal interlocking screw while the wrist was in the cast. Complete healing of the radiocarpal joint was evident at four months postop. The screw was managed with observation because the patient was asymptomatic, and the screw was barely palpable. At 16 months postop, the screw position remained unchanged, and there were no signs of tendinitis or tendon injury (Figure 6).

Case 3

**Figure 7:**
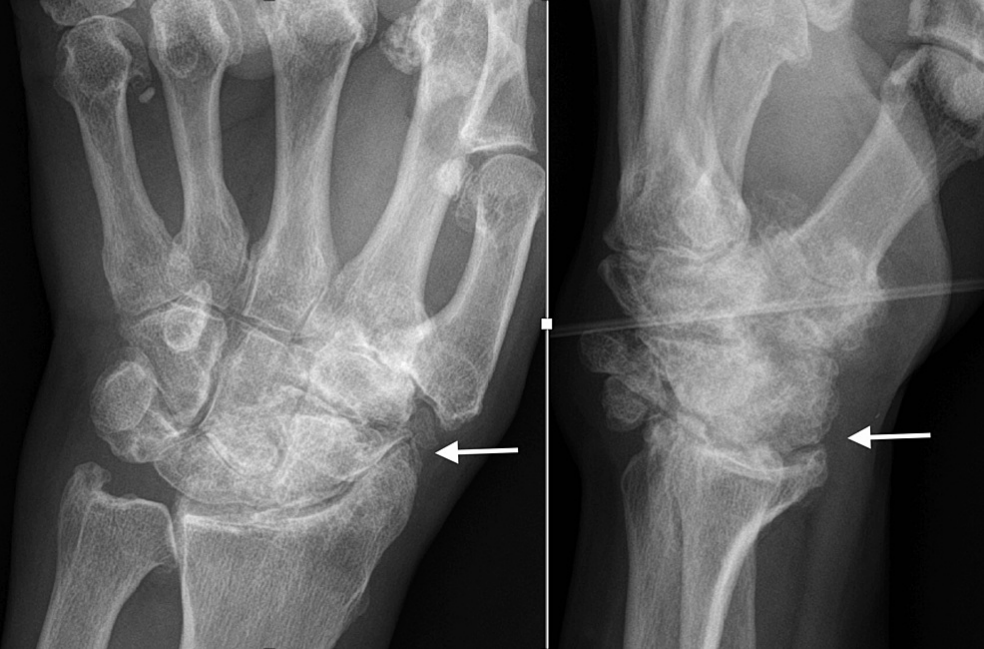
Preoperative radiographs of Case 3 A 73-year-old male with chronic advanced wrist arthritis. The white arrows show the radiocarpal and midcarpal arthritis.

A 73-year-old male, left-hand dominant, nonsmoker, with unremarkable past medical history except type II diabetes presented with chronic progressive left wrist pain, weakness, and motion limitation, which failed to respond to nonoperative management (Figure 7).

**Figure 8:**
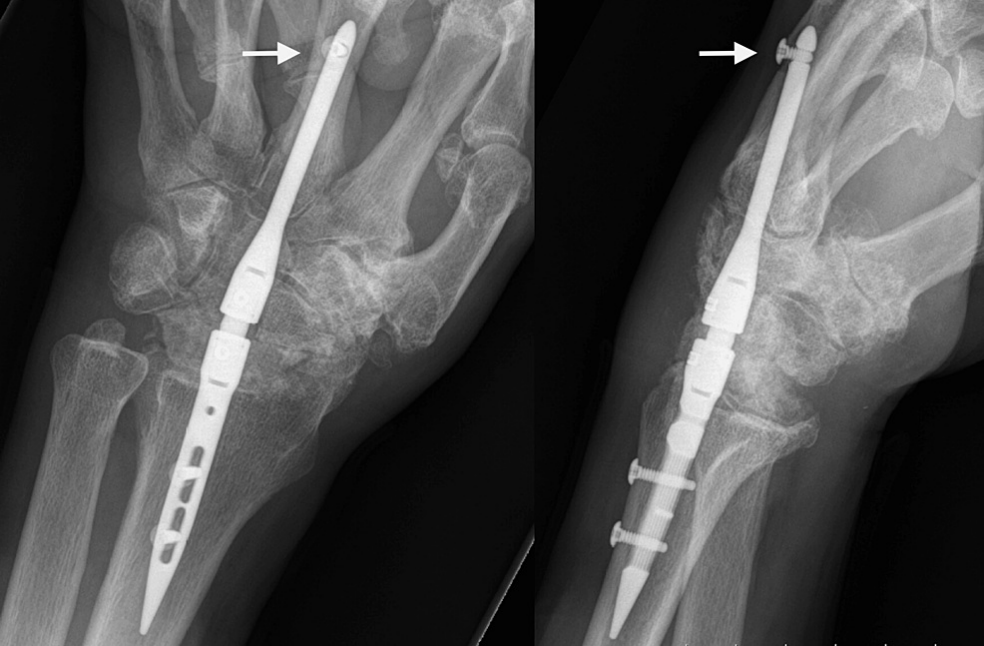
Immediate postoperative radiographs of Case 3 Posteroanterior and lateral views after total wrist arthrodesis using IMPLATE intramedullary nail and proximal row carpectomy. The white arrows show the proper positioning of the distal screw.

The patient underwent TWA for end-stage radiocarpal and midcarpal arthritis in March 2022 using intramedullary nail fixation (Figure 8).

**Figure 9:**
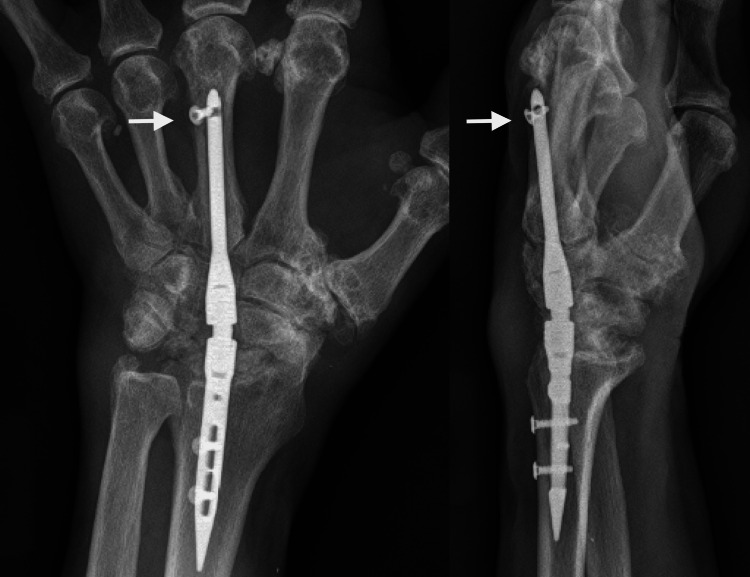
Follow-up radiographs of Case 3 Posteroanterior and lateral views at 3.5 months postop show that the distal unicortical screw backed out. The white arrows show the distal screw loosening and migration.

Proximal row carpectomy was used for the bone graft. He was placed in a custom fabricated wrist splint postoperatively for four months, and physical therapy for finger motions and forearm rotation was started at two months postop. At 3.5 months postop, progressive healing of the radiocarpal joint was evident. At this point, the distal interlocking screw showed evidence of radiographic loosening and backing out (Figure 9).

However, the patient was asymptomatic, and the screw was barely palpable. There was no clinical evidence of tendinitis; therefore, we decided to manage the loose screw expectantly with yearly follow-up.

## Discussion

Screw loosening is not uncommon with respect to orthopedic hardware [2]. It is typically caused by excessive motion at the screw/plate/bone interface, related to the normal micromotion of a fixation construct, or due to infection, nonunion, or periprosthetic fracture. Construct micromotion from small stress-dependent deflection of the implant (a function of the implant dimensions and modulus of elasticity) and the time-dependent strength of the implant/bone interface (a function of stress relaxation and creep) is expected in any fixation construct. This motion resolves once a fracture has healed or fusion is complete. Fixation constructs are designed to provide relative and not absolute stability as some degree or micromotion is desirable to achieve a bony union [6]. Too much motion, however, at a fracture or fusion site can lead to nonunion and subsequent implant failure.

Examining the mechanics of the distal fixation of the IMPLATE nail, we see the device functions differently than most typical long bone nails as it relies on compression of the nail to the third metacarpal endosteum by means of a unicortical screw to achieve stability. Femur and tibial nails, for example, do not compress against the endosteum and require bicortical fixation with a minimum of two cortices through a threaded locking or nonlocking hole in the nail. For the IMPLATE distal nail, the proximally threaded unicortical screw threads into the nail to achieve fixation. By virtue of this design, unless the nail is already fully compressed to the bone surface, the threads in the bone will necessarily be stripped to allow the nail to compress against the bone, thus losing the point of fixation in the bone. Any repetitive toggle motion of the screw within the nail could cause it to back out. Presumably, the designers avoided bicortical fixation to minimize the risk of finger flexor tendon injury.

We consider three possibilities contributing to the distal screw loosening. First, the third carpometacarpal (CMC) joint is a potential site of motion, despite it being a plane-type joint with relative stability. We speculate that tightening the distal screw might force the metacarpal into flexion from the CMC joint rather than pulling the nail up against the endosteal surface. Therefore, the dorsal CMC joint ligaments are loaded like a stretched rubber band leading to screw loosening when the energy is discharged. It is reasonable to disrupt the dorsal CMC ligaments as part of the IM nail surgical technique and incorporate this joint in the fusion mass.

Second, micromotion of the fixation construct at the bone/implant interface may contribute to screw loosening. Compared to plate fixation, an IM nail construct generally has more inherent motion at the bone-implant interface as well as the fracture or fusion site [7]. With this motion, the see-saw effect causes the most stress at the furthest point on the lever arm, which is the distal locking screw. Moreover, any bony resorption related to focal stress at this single point of distal fixation will lead to more motion and loosening.

Third, based on orthopedic surgery principles, the number of engaged cortices plays a role in the construct stability. Even though a tight isthmus fit is considered one point of fixation for an IM nail, the addition of a single unicortical screw would not be expected to provide any appreciable stability. IM nails generally require a minimum of two cortices of fixation on either side of the fracture.

The exact reason for the device failure in our patients could be related to any or all of the reasons speculated above. We did not include the third CMC joint as part of the fusion mass in any of our cases. Though it is not explicitly indicated in the implant technique guide, some authors recommend including the second and third CMC joints in the fusion mass when using a spanning plate to prevent motion at that joint, potentially leading to plate failure [8]. CMC joint nonunion is reported in about 19% of the spanning plates in which CMC fusion is attempted [1]. This complication can be avoided using a “T” plate that stops short of the CMC joint and has demonstrated radiocarpal fusion rates similar to spanning plates [9]. However, even with a non-spanning “T” plate, the removal rate is 13% (two out of 15 patients) in one study [9]. A retrospective review of spanning plates used for radiocarpal fusion reported a reoperation rate of almost 20% for symptomatic hardware [10]. Postoperative complications after TWA with plates, including plate prominence or failure, screw loosening, tendon adhesions, nonunion, and infection, are as high as 30%-60%, and the reoperation rate for any reason varies from 19% to 64% [10,11].

Orbay et al. reported the surgical technique for TWA using a locked IM nail in 2012 [5]. They also reported the results of seven patients with 100% union between six and 12 weeks. Notably, the third CMC joint was incorporated into the fusion mass in all patients. In their series, there was no need for implant removal or implant-related complications requiring reoperation [5]. Walker et al. reported their early experience with IMPLATE nails in 2021, achieving union in eight out of nine patients [12]. They reported three out of nine distal screws backed out. The first patient developed pain over the distal screw two months postop and had the screw removed. The same patient required revision CMC fusion because of a symptomatic nonunion at seven months postop. In the other two patients, screw back out was evident at five weeks postop, with one requiring screw revision and one observed because of asymptomatic migration [13]. The surgical technique followed the prior description; however, the details of the third CMC incorporation in the fusion mass were not provided. They also reported one metacarpal fracture with subsequent radiocarpal nonunion requiring revision.

Another report of using a non-locked IM nail with Wrist Fusion Rod (Nakashima Medical, Okayama, Japan) augmented by other fixation for radiocarpal fusion in six wrists of four patients with rheumatoid arthritis showed 100% union [14]. The technique includes inserting a single rod in the distal radius and then advancing it into the carpus and third metacarpal. The rod is bent in situ using a rod bender to achieve appropriate dorsiflexion and ulnar deviation position. It is fixed distally using a single bicortical screw in the metacarpal adjacent to the nail to avoid rotation. The two radiographic complications, including rod fracture and nondisplaced proximal metacarpal fracture, were managed nonoperatively, and both were attributed to motion at the third CMC joint [14].

Plate failure for the wrist fusion occurs mainly at the distal screws, which is attributed to the third CMC nonunion and excess motion [10]. Moreover, loosening of a total wrist arthroplasty commonly occurs at the distal screw and pegs in the carpal and metacarpal [15]. It implies that the metacarpal shaft might not be biomechanically compatible with IM nails, or perhaps, a press-fit/porous-coated nail would withstand the forces better.

One advantage of the IMPLATE IM nail over plates is the possibility of adjusting the plane of the wrist in both extension and radioulnar deviation using the connectors with several angles. The IM nail provided enough stability to promote bone fusion as was evident in the excellent fusion rates [5,12,14]. Moreover, none of these papers reported soft tissue irritation and implant removal except for that related to distal screw migration. Compared with plate fixation with 11%-16% hardware removal rate [1,2], it is speculated that the locked IM nails substantially reduce reoperations after TWA, specifically for hardware removal. Although the limited data about the IM nail has shown 0% nail removal, there was a 13% (two out of 16) rate of distal screw removal [5,12]. We should also take into consideration that extracting IM nails from the wrist requires taking down the fusion mass, and most surgeons would undoubtedly be hesitant to proceed unless absolutely necessary, such as in the case of periprosthetic infection.

## Conclusions

In conclusion, the most frequently reported complication with IM nails is distal screw migration, which can be managed nonoperatively in most cases; however, the long-term outcome of expectant management is unknown. We also recommend incorporating the third CMC joint in the fusion mass at the time of the index surgery as this could be a source of excess motion causing screw loosening. As this implant appears to have many benefits over plate fixation, second-generation designs will hopefully include more bicortical fixation points to address distal screw loosening.
